# Influence of headache pain intensity and frequency on migraine-related disability in chronic migraine patients treated with OnabotulinumtoxinA

**DOI:** 10.1186/s10194-020-01157-8

**Published:** 2020-07-11

**Authors:** Marta Torres-Ferrus, Victor José Gallardo, Alicia Alpuente, Patricia Pozo-Rosich

**Affiliations:** 1 Headache and Craniofacial Pain Unit, Neurology Department, Hospital Universitari Vall d’Hebron, Barcelona, Spain; 2 Headache and Neurological Pain Research Group, Vall d’Hebron Research Institute, Barcelona, Spain

**Keywords:** Migraine, Preventive treatment, Headache, Outcome measures, Effectiveness, Onabotulinumtoxin A

## Abstract

**Background:**

There is a need to establish which are the more relevant headache-related outcomes that have an impact on our patient’s lives to accurately evaluate treatment response in daily clinical practice.

**Objective:**

The aim of this study was to evaluate the relevance of clinical trial endpoints in clinical real-life disability improvement in response to migraine preventive treatment with OnabotulinumtoxinA.

**Methods:**

This is an observational prospective study. We included patients with chronic migraine fulfilling ICHD-3beta/3 criteria. We prospectively collected data of 8 headache-related and acute medication use endpoints recommended by the Guidelines of the International Headache Society for controlled trials of preventive treatment of chronic migraine. We evaluated their impact on disability improvement after 6 months of treatment with OnabotulinumtoxinA. We defined as a responder in disability, patients with ≥50% MIDAS score reduction after 2 cycles of treatment following PREEMPT protocol. We performed an analysis to measure the impact of improvement in the evaluated outcome measures according to perceived disability in clinical practice.

**Results:**

We included 395 patients (85.1% women, mean age 46.7 ± 12.6 years). Mean headache frequency at baseline was 26.5 ± 5.2 headache days/month. After 6 months, 49.1% of patients were headache-related disability responders. From all outcome measures collected, variables independently associated to disability improvement were headache days reduction (*p* = 0.02) and ≥ 50% pain intensity reduction (*p* = 0.04). A ≥ 50% reduction in headache frequency or pain intensity showed similar influence on disability improvement after treatment.

**Conclusions:**

Headache pain intensity is as important as frequency when evaluating the clinical response and impact on patient headache-related disability after migraine preventive treatment with OnabotulinumtoxinA.

## Introduction

Chronic migraine, is a prevalent and highly disabling neurological disease [1]. Suffering recurrent migraine attacks produces an abrupt and non-predictable functional disruption of daily life and entails an overwhelming decrease in the patient’s quality of life with a clear negative impact on a personal, family, working, social and economic level. As neurologists and headache specialists, in the absence of treatments that dramatically change the course of migraine, our main goal when treating a patient with chronic migraine is to recognize and minimize headache-related disability.

OnabotulinumtoxinA is an effective chronic migraine preventive therapy that improves disability by reducing quantifiable outcome measures, as headache frequency, duration or attack intensity [2, 3]. Additional benefits may include an enhancement of response to acute treatment, together with a reduction in acute medication intake [4]. As with other migraine preventive treatments, the use of OnabotulinumtoxinA may decrease visits to outpatient clinics, emergency departments and neuroimaging tests proving to be cost-effective [5, 6]. Hence, all these clinical and economical potential effects should be considered when we evaluate treatment effectiveness. Nowadays, due to the lack of quantifiable biochemical or image-based biomarkers in migraine treatment response evaluation, information taken from clinical interview is still essential. However, we need to establish which are the most relevant headache-related outcomes that have an impact on our patient’s lives to accurately evaluate treatment response in daily clinical practice. An inaccurate evaluation of therapy effect may lead to discontinuation of treatment in patients who could be experiencing a substantial benefit in terms of reduction of disability or *viceversa*, lead to continuation of a therapy that is not producing the expected improvement increasing unnecessary costs.

Recently published International Headache Society Guidelines for clinical trials of preventive treatment of chronic migraine [7] have set the recommendations on which primary or secondary endpoints should be used. Following these guidelines, the primary endpoint for clinical trials in migraine prevention should be either a change in migraine days; a change in moderate to severe headache days; or responder rate. However, other outcomes frequently used as secondary endpoints in clinical trials (e.g. headache intensity, headache free days, duration of effect, use of acute treatment or headache-related disability) are not just indirect measures of efficacy in real-life clinical setting. An improvement in what we consider secondary outcome measures in clinical trials can have a key impact in headache-related disability reduction and quality of life improvement in our patients. So, in our every-day clinical practice, we need to figure out which of these outcome measures for clinical trials can better evaluate the impact of the preventive treatment on patient’s quality of life.

The aim of this study was to evaluate which outcome measures recommended for clinical trials of chronic migraine preventive treatment have a greater influence and better reflect the response to treatment with OnabotulinumtoxinA in regards to patient’s disability.

## Methods

### Data collection

This is a prospective observational study. We consecutively included patients with migraine fulfilling the International Headache Disorders Classification 3/3beta criteria [8, 9] from July 2014 to October 2019, who had the indication to start a migraine preventive treatment. We included chronic migraine patients at first-time treatment with OnabotulinumtoxinA [10]. We did not exclude patients with stable dose of concomitant oral preventive medications.

Demographic data, comorbidities and migraine characteristics were collected at baseline and after two cycles of OnabotulinumtoxinA following PREEMPT protocol [11]. We included in the evaluation eight headache-related and acute medication-intake endpoints recommended in the Guidelines for clinical trials [7]: change in headache days, > 50% headache days responder rate, change in moderate to severe headache days, > 50% moderate-severe headache days responder rate, conversion to episodic migraine, > 50% intensity of headache responders rate, acute treatment utilization (number of tablets/month) and conversion of medication overuse to non-medication overuse. Patients recorded in a diary headache frequency (total headache days and moderate-severe headache days) and acute medication used for headache relief. A headache day was defined as any day with headache that lasted at least 4 h, and a moderate-severe headache day was defined as a day with moderate or severe pain that lasted 4 h at least [7]. The baseline evaluation included mean headache frequency and analgesic use during 3 months prior to inclusion and we calculated the percentage reduction of these mentioned categories after the second cycle of OnabotulinumtoxinA (from month 4 to 6). Patients also subjectively rated headache pain intensity reduction compared to baseline in three categories (≤25%, 25–49%, ≥50%). Finally, patients completed the Migraine Disability Assessment (MIDAS scale score and grade) [12].

The MIDAS questionnaire is a widely used validated scale designed to evaluate disability within the last 3 months. The patient evaluates, in a 5-item questionnaire, the reduction in the performance, in days, of work/school, household work, and family/social activities. A score 0–270 is used to indicate the overall level of disability due to headaches and the final score indicates the level of disability which can be then classified into four categories [12]. Scores on the MIDAS are highly correlated with physician judgments regarding patients’ pain, disability, and need for medical care [13] and changes in the MIDAS score may serve as an endpoint in assessing treatment efficacy [14]. The MIDAS questionnaire may have some advantages over other headache-related disability scales in this study. A proportion of patients treated with OnabotulinumtoxinA may experience a wearing off of the effect during the last weeks the treatment cycle with a clinical worsening [15], so other headache-related disability scales with shorter recall period could underestimate treatment response. In order to focus our interest on change in disability after treatment and evaluate the impact of the other outcome measures on disability, we decided to consider a ≥ 50% MIDAS score reduction as a main treatment response variable in our analysis.

The study was approved by Vall d’Hebron Ethics Committee. All patients gave their written informed consent for data collection.

### Statistical analysis

Descriptive and frequency statistics were obtained and comparisons made using the R software version (3.6.1). For nominal variables, measures of frequencies used are counts (n) and percentages (%). For quantitative variables, central tendency used is the mean and the standard deviation (SD) as a measure of dispersion (mean ± SD). Statistical significance for intergroup variables was assessed by Pearson chi-square or Fisher’s exact test for categorical variables, the linear trend chi-square test for ordinal variables and the independent t-test Student’s t test or Mann-Whitney U test for quantitative variables. Paired t-test or Wilcoxon signed rank test were used to assess pre-post changes in numerical variables related to the main treatment effectiveness. McNemar’s test for paired proportions was used for categorical data. Parametric tests were only used when a normal distribution was assumed. Data normality were verified through visual methods (histograms and Q-Q plots) and normality test (Kolmogorov-Smirnov test). From the univariate analysis, significant clinical outcomes measures associated to ≥50% MIDAS score reduction were identified.

A logistic regression analysis with backward elimination approach was performed to identify patients with a ≥ 50% MIDAS score improvement as a main response variable to evaluate migraine preventive treatment. Data was initially partitioned into training and testing sets and a 10-fold cross-validation (10-fold CV) was also performed in order to validate the prediction outcomes of our logistic classifier, assessing the model performance by their accuracy and Cohen’s Kappa. Multicollinearity between potential predictors was assessed with the calculation of correlation matrix and the phi correlation coefficient. Hosmer-Lemeshow test was also computed in order to assess the goodness of fit of the model computed. Finally, a receiver operating characteristic curve (ROC) was performed with the predicted probabilities from the model in order to evaluate its discrimination ability based on disability improvement. The logistic regression analysis and the ROC calculation was performed with the caret (6.0–85) and ROCR (1.0–7) packages of R.

*P*-values presented are for a two-tailed test and *p*-values < 0.05 were considered as statistically significant. No statistical power calculation was conducted prior to the study because sample size was based on available data.

## Results

We included in the analysis 395 patients (85.1% women, mean age 46.7 ± 12.6 years), that represents the 86.8% of patients that initiated treatment with Onabotulinumtoxin A during de inclusion period (5.9% of patients were excluded due to missing information and 7.3% were lost of follow up). From these, mean headache frequency at baseline was 26.5 ± 5.2 headache days/month and 61.3% (242/395) fulfilled criteria for medication overuse. Table 1 shows main demographic and migraine characteristics at baseline.

**Table 1:** Baseline Demographics and Migraine Characteristics

	***N*** = 395
Gender (female)	85.1%
Age (mean, years ± SD)	46.7 ± 12.6
Aura	28.6%
Migraine duration (mean, years ± SD)	28.6 ± 15.0
Chronification time (mean, years ± SD)	10.5 ± 9.9
Headache frequency (days/month ± SD)	26.5 ± 5.2
Moderate-Severe headache frequency (days/month ± SD)	12.7 ± 7.5
Moderate-Severe headache monthly rate (% ± SD)	49.0 ± 27.6
Acute treatment (% of use)	95.7%
NSAIDs	54.6%
Triptans	46.1%
Other analgesics	36.3%
Analgesic use (mean, tablets/month ± SD)	42.2 ± 42.4
Daily use of analgesic	38.7%
Medication overuse	61.3%
Stable concomitant preventive treatment (%)	77.0%
Neuromodulators	39.2%
Betablockers	14.9%
Antidepressants	61.5%
Others	10.6%
MIDAS disability category	
None	5.6%
Mild	4.8%
Moderate	6.8%
Severe	82.8%
MIDAS score (mean ± SD)	82.6 ± 64.6

Abbreviations: *SD* standard deviation, *NSAID* Nonsteroidal anti-inflammatory drugs, *MIDAS* Migraine Disability Assessment

After 2 cycles of OnabotulinumtoxinA, 49.1% (194/395) of patients reduced their MIDAS score in ≥50% and were considered disability responders. Table 2 shows the change in all outcome measures evaluated. There was a statistically significant reduction in headache frequency (26.5 ± 5.2 to 15.2 ± 10.1 headache days/month, *p* < 0.001) with 51.4% (203/395) of patients experiencing a ≥ 50% frequency reduction. Moderate to severe headache days were decreased from 12.7 ± 7.5 to 6.7 ± 6.0 days/month (*p* < 0.001) and 54.2% (214/395) showed a ≥ 50% reduction in moderate to severe days. The headache pain intensity reduction was rated as ≥50% in 35.8% (141/395) of patients and analgesics intake was cut in half in 60.4% (239/395) of patients. A 52.9% (209/395) of patients improved and shifted from chronic to episodic migraine and 37.5% (148/395) stopped overusing acute headache medication.

**Table 2:** Change in evaluated clinical outcome measures after treatment

	All sample (***N*** = 395)	Responders^**a**^ (***N*** = 194)	No responders (***N*** = 201)	***P***-value^**†**^
Headache days/month	−11.3 ± 10.7	−15.3 ± 10.2	−7.4 ± 7.3	< 0.0001
Moderate-severe headache days/month	−7.3 ± 7.0	−8.5 ± 8.9	−3.6 ± 8.1	< 0.0001
≥50% headache days rate	51.4%	66.5%	36.8%	< 0.0001
≥50% moderate-severe headache days rate	54.2%	70.6%	38.3%	< 0.0001
Conversion to episodic migraine	52.9%	68.0%	38.3%	< 0.0001
≥50% pain intensity reduction	35.8%	50.0%	21.0%	< 0.0001
Acute treatment use (pills/month)	−24.9 ± 26.5	−34.2 ± 52.7	−14.1 ± 37.9	< 0.0001
≥50% acute treatment reduction (pills/month)	60.4%	69.6%	49.3%	< 0.0001
Conversion from medication overuse to non-medication overuse	37.5%	44.8%	30.3%	0.004
≥50% MIDAS score reduction rate	49.1%	*NA*	*NA*	*NA*

^a^Responders were patients who presented a ≥ 50% improvement in MIDAS score after two cycles of OnabotulinumtoxinA. †*P*-values are assessed between responders and non-responders. Unpaired t-test were used for quantitative variables and Pearson’s Chi-squared test with Yate’s continuity correction for dichotomous variables

### Clinical endpoints associated to disability improvement

We did not find statistically significant differences in demographic and basal characteristics between responders and non-responders based on ≥50% in MIDAS score reduction.

In regards to established Guideline preventive treatment trial endpoints shown in Table 2, the univariate analysis revealed that all outcome measures were statistically significantly associated to a response in disability (≥50% MIDAS score reduction). However, only the mean reduction in headache days (OR [95%CI]: 0.981 [0.900–0.989]; *p* = 0.016) and ≥ 50% the reduction in headache intensity (OR [95%CI]: 2.486 [1.422–4.391]; p = 0.016) resulted in statistically significant outcomes associated independently to improvement in disability after two cycles of OnabotulinumtoxinA in the training set (see Table 3). The accuracy of the model (95% CI) in the training set was 0.68 (0.62–0.73), kappa = 0.36 and *p*-value < 0.001; in the testing set, this model was able to classify patients according to their improvement in disability with an accuracy of 0.64 (0.62–0.75), kappa = 0.30 and p-value = 0.015. Finally, the area under the ROC curve (AUC) was used to characterize how well the model was able to discriminate ≥50% MIDAS improvement based on the independently associated clinical outcomes. The AUC of this model was 0.77 ± 0.032 (standard error), with sensitivity of 72.50% and a specificity of 65.3%.

**Table 3:** Logistic regression model for ≥50% MIDAS score reduction rate

	OR (95% CI)	***P***-value
**Headache days/month reduction**	**0.944 (0.900–0.989)**	**0.016**
Moderate-severe headache days/month reduction	0.981 (0.948–1.014)	0.259
≥ 50% headache days rate	0.863 (0.296–2.523)	0.785
≥ 50% moderate-severe headache days rate	0.811 (0.798–1.348)	0.635
Conversion to episodic migraine	1.014 (0.490–3.742)	0.976
**≥ 50% pain intensity reduction**	**2.486 (1.422–4.392)**	**0.002**
Acute treatment use (pills/month)	0.993 (0.982–1.001)	0.144
≥ 50% acute treatment reduction (pills/month)	0.848 (0.417–1.689)	0.644
Conversion to non-medication overuse	1.073 (0.392–2.591)	0.814

Odds Ratio estimated from logistic regression analysis of the clinical outcomes associated to disability improvement. In bold are statistically significant outcomes from the model. The accuracy of the model (95% CI) in the testing set (*n* = 77 patients) was 0.64 (0.62–0.75), kappa = 0.30 and *p*-value = 0.015. *OR* Odds-ratio, *CI* Confidence Interval

### Primary clinical endpoints redefinition: frequency and intensity

**Fig. 1:**
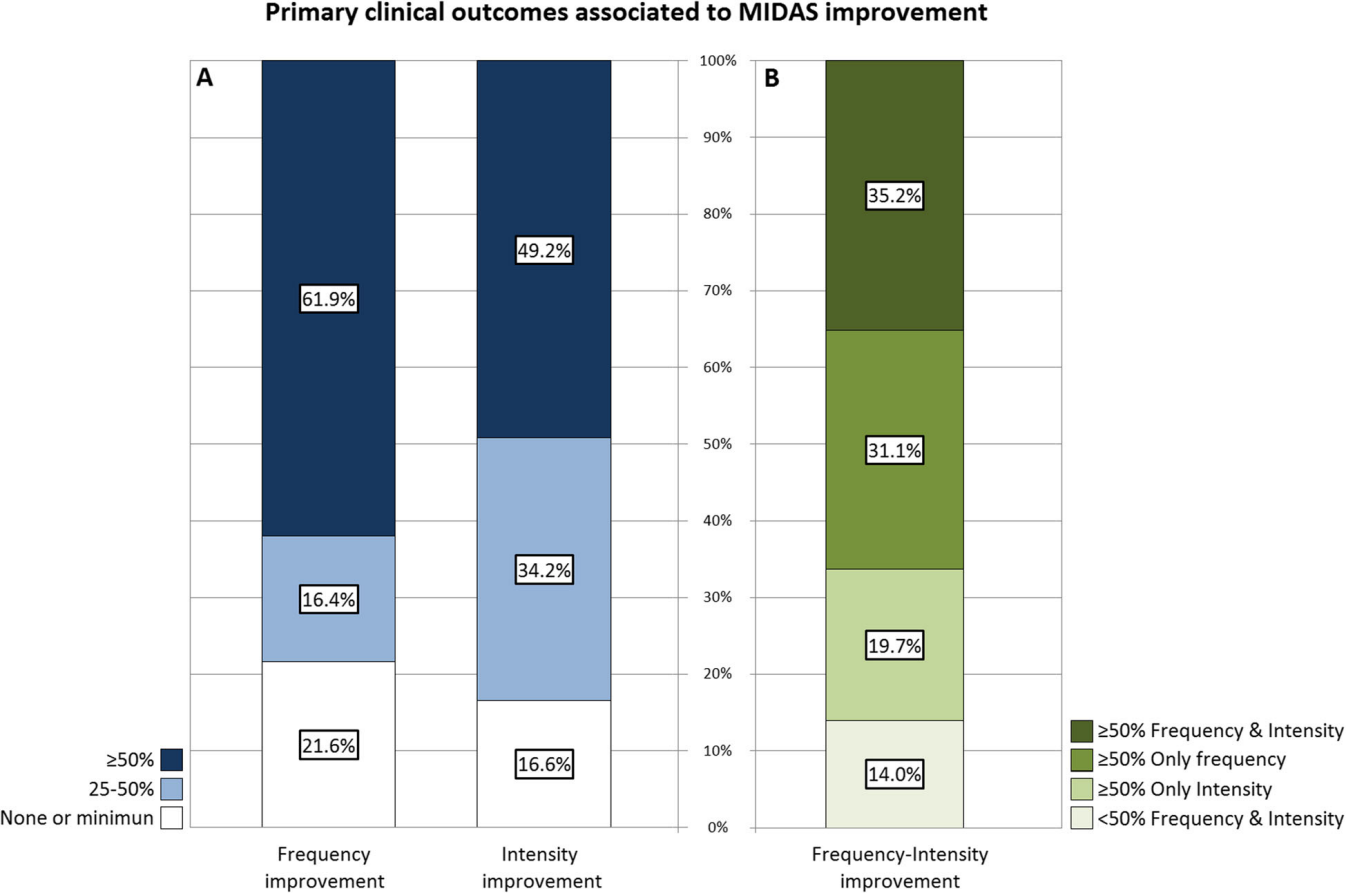
Clinical outcomes that showed significant association with ≥50% MIDAS score reduction after 6 months of preventive treatment with OnabotulinumtoxinA. **a** shows response rate in frequency, intensity, and (**b**) co-variable frequency-intensity according to treatment response

Frequency-based outcomes are considered a primary endpoint for treatment response evaluation in clinical trials. However, our analysis showed a similar influence of frequency and intensity improvement on treatment response when considering a ≥ 50% MIDAS improvement: the 50.0% of patients who had a ≥ 50% pain intensity improvement, had also a ≥ 50% MIDAS improvement (*p* < 0.001). Similar proportion was observed for headache frequency: the 66.5% of patients who had a ≥ 50% frequency improvement, had also a ≥ 50% MIDAS improvement (*p* < 0.001) (Fig. 1a). Because of these similar proportions, we decided to further study on this.

In our cohort, 19.7% were considered disability responders without achieving ≥50% frequency reduction (see Fig. 1b). A sub-analysis of this group showed that those patients who had a ≥ 50% intensity reduction but did not experience ≥50% frequency improvement had a 62.8% chance of improvement in disability, with a mean MIDAS score reduction of 66.2 ± 69.6 points. *Viceversa*, this response probability is similar to the one seen in patients with ≥50% frequency reduction but poor effect on intensity (57.7%), with a mean reduction of 51.6 ± 54.6 points in MIDAS score. Although our data show a higher impact on disability when the improvement is driven by a decrease in intensity, the differences with the group which improves in frequency are not statistically significant. In this subset of patients with ≥50% intensity response, we did not find any demographical or clinical characteristic that predicted disability improvement.

Finally, a 35.0% of patients did not have a good response either in frequency or in intensity. In this group of patients, only 6.9% had ≥50% MIDAS improvement. When we analyzed this small group of patients, we observed that the disability improvement in this subgroup of was associated with a higher decrease in the severe days whether there were not significant changes on headache frequency (severe/headache days in < 50% MIDAS: − 20.3% ± 19.7 vs. ≥50% MIDAS: − 35.0 ± 34.99; *p* = 0.020), what also points to the influence of the intensity of headache.

## Discussion

Migraine is considered the second most disabling neurological disorder in years lived with disability [16]. Preventive treatment in chronic migraine can help reduce headache frequency or attack intensity and improve a patient’s quality of life [17]. Our research wants to serve as a reflection on which outcome measures used in clinical trials are more likely to have an impact on the patient’s disability improvement, and consequently to assess treatment response in a real-life clinical setting.

We demonstrate in a clinical sample of patients with chronic migraine that, after 6 months of treatment with OnabotulinumtoxinA, headache frequency reduction is not the only outcome independently associated with MIDAS improvement but also pain intensity, and should be considered also as a major clinical outcome measure. This study shows that patients without a significant improvement in headache frequency but who have an improvement in headache intensity have a similar impact on their disability, measured with MIDAS, as those who improve in frequency. Our results are in line with other studies which have tried to determine the influence of headache intensity and frequency on headache-related disability, and showed that disability increases progressively with increasing headache intensity but no significant relationship was found between headache frequency and disability [18]. So, a preventive treatment that has an impact on the severity of the attacks, reduces the patient’s disability in the same way as a reduction of ≥50% or more in headache frequency.

Although headache intensity is also considered a secondary outcome measure to evaluate preventive treatment response in clinical trials as well as clinical practice [7, 19], the vast majority of clinical real-life published studies that evaluate OnabotulinumtoxinA treatment response in different conditions, do not include the evaluation of headache pain intensity [20]. Besides, current clinical treatment guidelines for migraine prevention, define response to treatment based only in headache frequency reduction [21–23], even recommending to stop treatment if the frequency does not decrease in patients. Based on our findings, if we consider our patient’s disability, both frequency and intensity should be evaluated and considered, as it is common to find patients who experience a clear beneficial effect from a preventive treatment with just an improvement in the intensity or severity of attacks and without a notable influence on headache frequency.

Although our data show the importance of headache severity on patient reduction in disability, we have to keep in mind that changes in other measures, or the add up of several minor changes in more than one outcome measure may lead to a relevant global effect on a patient’s disability and quality of life. Acute medication intake and its overuse, is also a highly clinically relevant outcome and, in some patients, could turn into a key aspect in chronic migraine prevention. Besides, in a clinical setting, physicians should respect patient’s preferences. Patient’s preferences for a migraine preventive therapy include effectiveness but also tolerability, speed of onset, formulation, dosing and cost [24, 25] that should also be re-evaluated when treatment response is assessed in order to provide the patient with an effective and well tolerated treatment.

This study has several strengths including the longitudinal study design with prospective data collection in a large and well characterized sample of migraine patients that were evaluated by headache experts. The main limitation is that only response to preventive treatment with OnabotulinumtoxinA has been evaluated in this study and therefore, all patients included suffered from chronic migraine. However, a unified sample has some advantages for this analysis since it is a standardized treatment at per-protocol established doses that is administered in the clinic, which ensures a high rate of therapeutic compliance. Despite its limitations, our data make us reflect about the probable validity of clinical trial endpoints as well as real-life outcome measures in order to properly reflect the impact in a patient’s disability with preventive treatments for chronic and probably episodic migraine. Moreover, in our opinion, future studies in chronic and episodic migraine should evaluate the response to oral as well as calcitonin gene related peptide monoclonal antibodies taking into consideration both changes in frequency and intensity of migraine.

A point that needs further discussion is the use of headache-related disability, measured by MIDAS scale, as a principal outcome measure. As clinicians, our main goal when we treat any headache patient should always be to minimize headache-related disability and its impact on quality of life. We consider that, despite its limitations, the MIDAS scale is a global, well-validated and widely-used measure of migraine-related disability [26, 27]. Indeed, the other widely used scale to assess headache-related disability is Headache Impact Test 6 or HIT-6 [28]. There is scarce comparative data on these two scales, showing a correlation between MIDAS and HIT-6 scores, especially for chronic migraine patients [29–31]. Compared to MIDAS, HIT-6 scale includes a more emotional and subjective evaluation of disability and appears to be more influenced by headache intensity while MIDAS score could be more influenced by frequency [30]. We also tested our findings using HIT-6 scale in a smaller sample (210 patients) and although there was also a statistically significant reduction of mean HIT-6 before and after treatment associated to frequency and intensity improvement, the mean change in the numerical score did not allow us to test the statistical model.

## Conclusions

Intensity is as important as frequency when evaluating the improvement in disability in chronic migraine patients after preventive treatment with OnabotulinumtoxinA. A 50% reduction in headache frequency and intensity could equally and individually be considered as primary outcome measure. In future clinical preventive migraine treatment trials, we might consider the importance of reflecting the change not only in headache frequency but also in headache pain intensity as a primary outcome measure which is meaningful for patients, as it has an impact on their migraine related disability.

## Data Availability

The datasets used and/or analysed during the current study are available from the corresponding author on reasonable request.

## Abbreviations

AUC: Area Under the Curve
CI: Confidence Interval
CV: Cross Validation
HIT-6: Headache Impact Test 6
MIDAS: Migraine Disability Assessment Test
OR: Odds Ratio
ROC: Receiver Operating Characteristic Curve
SD: Standard Deviation
